# Supplementary material for: Benchmarking Data Sets from PubChem BioAssay Data: Current Scenario and Room for Improvement
Source: Int J Mol Sci. 2020 Jun 19;21(12):4380. doi: 10.3390/ijms21124380 (PMC7352161; doi:10.3390/ijms21124380)
Supplement: Supplementary file 1 [file ijms-21-04380-s001.pdf]

Supplementary Materials

Benchmarking Data Sets from PubChem BioAssay Data: Current Scenario and Room for Improvement

Viet-Khoa Tran-Nguyen [1] and Didier Rognan [1,*]

[1] Laboratoire d'Innovation Thérapeutique, UMR7200 CNRS-Université de Strasbourg, 67400 Illkirch, France; vktrannguyen@unistra.fr (V.-K.T.-N.); rognan@unistra.fr (D.R.)

* Correspondence: rognan@unistra.fr; Tel.:+33-3-68-85-42-35

Table S1. Number of PubChem bioactivity assays according to the number of tested substances, the number of active substances, the screening stage, and the target type. Statistics were updated as of April 30, 2020.

**Criteria**	**Assay type**	
	**Small-molecule assay**	**RNA interference assay**
**1. Number of tested substances (N_t):**		
• $N_t < 100$	1,060,707	22
• $100 \le N_t < 1,000$	4,530	92
• $1,000 \le N_t < 10,000$	1,359	14
• $10,000 \le N_t < 100,000$	422	48
• $N_t \ge 100,000$	701	1
**2. Number of active substances (N_a):**		
• $N_a < 10$	1,000,714	28
• $10 \le N_a < 50$	60,328	57
• $50 \le N_a < 100$	3,562	18
• $100 \le N_a < 1,000$	2,399	60
• $N_a \ge 1,000$	716	14
**3. Screening stage:**		
• Primary screening	1,416	113
• Confirmatory, dose-response curves not provided	276,216	0
• Confirmatory, dose-response curves provided	3,904	0
• Summary	701	10
• Screening stage not annotated	785,482	54
**4. Target type:**		
• Single protein	238,096	0
• Single gene	17	0
• Single nucleotide	95,325	0
• Multiple proteins	25,649	0
• Multiple genes	3	0
• Multiple nucleotides	8,646	0
• Protein-protein interaction	210	0
• None	795,301	177
**All**	1,067,719	177

Table S2. Number of compounds featured in PubChem bioactivity assays that satisfy each criterion of the Lipinski's rule of five, the Ghose filter, and the Veber's rule. Statistics were updated as of April 30, 2020.

Criteria	Number of PubChem compounds
**1. Lipinski's rule of five:**	
● Molecular mass ≤ 500 Da	88,667,112
● ClogP ≤ 5	78,183,471
● Number of hydrogen bond donors ≤ 5	101,211,514
● Number of hydrogen bond acceptors ≤ 10	99,344,677
● Compounds satisfying all criteria	73,062,126
**2. Ghose filter:***	
● Molecular mass from 180 Da to 480 Da	82,926,795
● AlogP from -0.4 to +5.6	79,473,661
● Number of atoms from 20 to 70	71,554,127
**3. Veber's rule:**	
● Number of rotatable bonds not exceeding 10	93,857,861
● Polar surface area not exceeding 140 $Å^2$	96,031,201
**All**	102,694,672

* The criterion regarding molar refractivity of the Ghose filter is not addressed in this table, as no relevant search option is available on PubChem Compound.

Table S3. Scaffold clusters of PubChem BioAssay active ligands (AID 493208) and the number of their representatives before and after LIT-PCBA filters.

Scaffold cluster	Scaffold structure	Number of substances falling into each scaffold cluster	
		Full data from AID 493208	Data from the LIT-PCBA MTORC1 ligand set
1	(O=C1CNCCO1)	3	1
2	(c1ccncc1)	38	9
3	(o1cccc1)	9	5
4	(O=C1CC=CN1)	15	9
5	(N=C1NC=CN1)	8	5
6	(c1nnn[nH]1)	4	1
7	(c1cncnc1)	40	4
8	(c1ccsc1)	3	0
9	(C1COCO1)	1	1
10	(O=C1NC=CC1=O)	5	5
11	(c1nc[nH]n1)	4	1
12	(O=C1NC=CSC=C1)	1	1
13	(C1CN=CN1)	1	0
14	(c1ccccc1)	9	2
15	(C1CC=CCN1)	5	2
16	(c1cn[nH]c1)	20	1
17	(c1cc[nH]c1)	13	4
18	(C1CCNCC1)	6	1
19	(c1nnc[nH]1)	5	2
20	(c1cscn1)	16	5
21	(C1OC=CO1)	5	3
22	(C1CNC=CC1)	2	0
23	(C1CCC\C=C/CC1)	2	1
24	(c1c[nH]cn1)	13	3
25	(C1CNCCN1)	13	3
26	(o1cccn1)	3	2
27	(C1COC=CC1)	1	1
28	(c1c[nH]nn1)	2	1
29	(C1C=CNC=N1)	1	0
30	(O=C1NC=CC=C1)	7	4
31	(C1COC=CO1)	3	1
32	(C1CC=CN1)	3	2
33	(C1CCC=NCC1)	1	1
34	(O=C1NN=CC=C1)	8	3

35	(c1ccnnc1)	4	1
36	(O=C1NC=CN=C1)	2	1
37	(C1NC=CN=C1)	2	0
38	(C1COC=CN1)	1	0
39	(O=C1NN=CN=C1)	3	0
40	(O=S1(=O)NC=CC=C1)	1	1
41	(o1ccnc1)	21	1
42	(c1nncs1)	4	2
43	(O=C1NC=CC(=O)N1)	5	1
44	(C1CC=CO1)	5	2
45	(C1OC=CC=C1)	1	0
46	(O=C1C=CNC=C1)	1	0
47	(O=C1NC=CS(=O)(=O)C=C1)	1	0
48	(C1CN=CO1)	3	0
49	(O=C1C=COC=C1)	1	1
50	(O=C1NC=NC=C1)	2	1
51	(c1ncncn1)	5	0
52	(c1cnncn1)	1	1
53	(C1CCC=CCC1)	2	0
54	(c1cn[nH]n1)	1	0
55	(C1CCCCC1)	1	0
56	(O=C1NCC=C1)	1	0
57	(C1CN=CC=C1)	2	0
58	(S1C=CC=NC=C1)	1	0
59	(o1nccn1)	1	1
**All**		**342**	**97**

Table S4. Scaffold clusters of PubChem BioAssay inactive ligands (AID 493208) and the number of their representatives before and after LIT-PCBA filters.

Scaffold cluster	Scaffold structure	Number of substances falling into each scaffold cluster	
		Full data from AID 493208	Data from the LIT-PCBA MTORC1 ligand set
1	(c1ccc2ncccc2c1)	638	288
2	(O=S(=O)(NC1=NCNCN1)c2ccccc2)	5	5
3	(O=C1NC=Cc2ccccc12)	196	195
4	(C1OC=Cn2ccnc12)	12	12
5	(O=C1NC=Nc2ccccc12)	256	150
6	(C1OC=Cc2ncccc12)	28	5
7	(O=C1NC(=O)c2cccnc2N1)	160	160
8	(O=C1NNc2ncccc12)	49	48
9	(O=C1OC=Cc2ccccc12)	34	25
10	(c1cnc2ccnn2c1)	439	305
11	(O=C1COc2ccccc2N1)	402	393
12	(c1ccc2[nH]ccc2c1)	1139	733
13	(O=C(CN1CCCNCC1)Nc2ccccc2)	8	8
14	(O=C1C=CNc2ccnn12)	35	31
15	(C1CCc2sccc2C1)	319	180
16	(C1COc2ccccc2O1)	281	250
17	(c1ccc2ncncc2c1)	98	16
18	(O=C(CC1NCCNC1=O)Nc2ccccc2)	19	19
19	(C1N=CNc2nccn12)	10	10
20	(c1nnc2cc[nH]c2n1)	33	27
21	(O=C(NC1C=CNC1=O)c2ccccc2)	13	13
22	(O=C1CC2=C(N1)NC(=O)NC2=O)	42	42
23	(o1cnc2ncccc12)	72	65
24	(O=C(CSC1=NC(=O)C=CN1)Nc2ccccc2)	15	15
25	(O=C1CNC(=O)N1c2ccccc2)	7	7
26	(O=C1NC(NS(=O)(=O)c2ccccc2)C(=O)N1)	15	15
27	(c1cnc2ncnn2c1)	274	205
28	(c1cn2nccc2nn1)	6	6
29	(O=C1NN=C2COC=CN12)	6	6
30	(O=C1CN=CN1C2CCCCC2)	5	0
31	(O=C(CC1NCCOC1=O)Nc2ccccc2)	7	7
32	(N=C1Nc2ccccc2S1)	8	8
33	(C1C=NNC1c2cn[nH]c2)	5	3

34	(O=C(CNS(=O)(=O)c1ccccc1)NCc2ccccc2)	14	14
35	(O=C1C=CN2C=CC=CC2=N1)	12	12
36	(O=C(CNS(=O)(=O)c1ccccc1)Nc2ccccc2)	46	39
37	(c1ccc(cc1)c2nn[nH]n2)	50	42
38	(O=C(CNc1ccccc1)Nc2ccccc2)	31	31
39	(c1ccc(cc1)n2cnnn2)	23	23
40	(O=C(CSc1nncs1)Nc2ccccc2)	34	34
41	(O=C1C2CN3CC1CN(C2)C3c4ccccc4)	26	23
42	(O=C1NCNc2ncccc12)	5	5
43	(o1ccc2ccccc12)	604	405
44	(C(N1CCNCC1)c2ccccc2)	21	21
45	(O=C1CCC2=C(O1)C=CNC2=O)	14	14
46	(c1ccc(cc1)c2ccc[nH]2)	139	100
47	(O=S(=O)(c1ccccc1)n2ccnc2)	14	14
48	(c1ccc2nsnc2c1)	175	170
49	(o1nc2ccccc2n1)	68	68
50	(O=C1NC(=NC=C1)SCc2oncn2)	3	3
51	(O=C(CCS(=O)(=O)c1ccccc1)Nc2ccccc2)	12	12
52	(C(Nc1ncc[nH]1)c2ccccc2)	9	8
53	(O=C1NC=C(C(=O)N1)S(=O)(=O)Nc2ccccc2)	5	5
54	(N=C1NC=Nc2[nH]ncc12)	27	27
55	(O=C(CCn1cnnn1)Nc2ccccc2)	13	13
56	(C1CN(CCN1)c2ccccc2)	166	151
57	(O=S(=O)(Nc1ncccn1)c2ccccc2)	27	27
58	(O=C1Nc2ncnn2C=C1)	12	12
59	(O=C1NC(=O)c2[nH]cnc2N1)	190	175
60	(O=C(CNCCc1ccccc1)Nc2ccccc2)	14	14
61	(O=C(NCCc1ccccc1)C(=O)Nc2ccccc2)	14	14
62	(C(c1nocn1)n2cccn2)	6	6
63	(N(c1ccccc1)c2nccs2)	17	14
64	(O=C(Nc1ccccc1)C2CNC(=O)C2)	11	11
65	(O=C(CSc1ocnn1)c2ccccc2)	8	8
66	(O=C1C=CN=C2C=CC=CN12)	438	424
67	(O=C(CSCc1cocn1)N2CCNCC2)	13	13
68	(O=C(Oc1ccn[nH]1)c2ccccc2)	42	15
69	(O=C1NC=CS(=O)c2ccccc12)	123	72
70	(O=C(CS(=O)Cc1cocn1)NCCc2ccccc2)	13	13
71	(O=C(CNc1ccccc1)NCCSCc2ccccc2)	14	14
72	(O=C(CSCc1cocn1)NCc2ccccc2)	13	13
73	(O=C1Oc2ccccc2C=C1)	438	336
74	(C(N1CCCCC1)c2cocn2)	50	44

75	(O=C(Nc1ccccc1)c2occc2)	30	26
76	(O=C(NC1CCNCC1)Nc2ccccc2)	12	12
77	(O=C1CSc2ccccc2N1)	138	134
78	(o1cnc2ccccc12)	528	287
79	(O=C1CCCC2=C1CC=CN2)	6	6
80	(c1cnn2cnnc2c1)	82	75
81	(c1ccc2[nH]cnc2c1)	682	415
82	(O=C(Nc1ccccc1)C2CCCN2)	5	5
83	(c1ccc2cnncc2c1)	88	40
84	(O=C1Cc2ccccc2N1)	126	124
85	(O=C1Nc2ccccc2O1)	359	354
86	(O=C1NN=C(C=C1)c2ccccc2)	70	67
87	(C1NC=Cc2ccnn12)	13	13
88	(O=S(=O)(NCC1CCCCC1)c2ccccc2)	18	12
89	(O=C1Nc2ccccc2N1)	128	128
90	(O=C1CN=Cc2ccccc2N1)	32	21
91	(O=C1C=CNc2ccccc12)	671	563
92	(O=C1NCC2=C1OC=CC2=O)	110	97
93	(C1Oc2ccccc2O1)	666	570
94	(O=C(NCc1cccs1)Nc2ccccc2)	17	15
95	(O=C(NCCc1ccccc1)c2occc2)	32	31
96	(O=S(=O)(c1ccccc1)c2c[nH]nn2)	15	15
97	(c1ccc2n[nH]nc2c1)	76	30
98	(O=C(Nc1ccccc1)\C=C\c2ccccc2)	21	19
99	(O=S(=O)(N1CCCCCC1)c2ccccc2)	11	11
100	(c1ccc(cc1)c2ccn[nH]2)	11	0
101	(N=C1NC(=O)CC(S1)C(=O)Nc2ccccc2)	8	8
102	(O=C1N=CNc2sccc12)	2	2
103	(O=C1CCC(=NN1)c2ccccc2)	48	48
104	(C1Cc2scnc2C=C1)	11	11
105	(C1C=Cc2ncsc12)	13	13
106	(O=C1CNc2ccccc2N1)	44	43
107	(O=C1CNCc2ccccc2N1)	70	63
108	(O=C1CCC2=C(COC2=O)N1)	12	12
109	(O=C1CCC2=C(O1)C=COC2=O)	7	7
110	(O=C(CCOc1ccccc1)Nc2ccccc2)	8	8
111	(c1ccn2ccnc2c1)	143	64
112	(O=C1CN(C2CCCCC2)C(=O)CN1)	50	41
113	(c1ccc(cc1)c2ccncn2)	11	11
114	(O=C(CSCc1ccccc1)NCc2ccccc2)	7	6
115	(C1CCc2[nH]ncc2C1)	13	13

116	(O=C1NC2=CC=CCC2=C1)	16	16
117	(O=C(NCc1ccccc1)c2occc2)	37	37
118	(o1ccnc1c2ccccc2)	32	32
119	(O=S(=O)(NCc1ccccc1)c2ccccc2)	12	5
120	(O=C(COc1ccccc1)Nc2cn[nH]c2)	6	6
121	(O=C(COc1ccccc1)Nc2cccs2)	14	14
122	(o1cnnc1c2ccccc2)	36	22
123	(O=C(Nc1ccccc1)c2c[nH]cn2)	5	5
124	(O=S(=O)(N1CCCCC1)c2ccccc2)	27	27
125	(O=S(=O)(N1CCOCC1)c2ccccc2)	27	27
126	(O=C(Nc1ccccc1)\C=C\c2occc2)	26	21
127	(O=C1NC(=O)c2c[nH]cc2N1)	59	42
128	(C1Cc2ccccc2CN1)	142	120
129	(C(Oc1ccccc1)c2occc2)	10	10
130	(C1CC2(CCN1)OCCO2)	34	31
131	(O=C(Nc1ccccc1)c2ccccc2)	133	76
132	(O=C(COc1ccccc1)NC2CCS(=O)(=O)C2)	12	12
133	(O=C1NC=CS(=O)(=O)c2ccccc12)	170	104
134	(O=C(NC1CCS(=O)(=O)C1)c2ccccc2)	32	32
135	(c1ccc(cc1)n2ccnn2)	20	20
136	(O=C1NN=Nc2sccc12)	80	34
137	(c1ccc2scnc2c1)	819	610
138	(O=C1NCc2cn[nH]c12)	118	91
139	(o1cnc(n1)c2ccccc2)	75	72
140	(O=C(NCC12CC3CC(CC(C3)C1)C2)c4cc[nH]n4)	6	0
141	(o1cnc(n1)c2ccncc2)	20	20
142	(c1ncc2cn[nH]c2n1)	225	175
143	(O=C(CSc1ocnn1)Nc2ccccc2)	36	36
144	(C1C=CNc2ncnn12)	273	164
145	(C(Oc1ccccc1)c2oncn2)	13	13
146	(c1ncc2cc[nH]c2n1)	76	3
147	(O=C1NN=Cc2ccccc12)	75	70
148	(O=C(CN1CCNCC1)Nc2ccccc2)	26	26
149	(c1cnc2sccc2c1)	168	111
150	(o1cnc(n1)c2occc2)	21	21
151	(O=C1NC(=O)c2ccsc2N1)	305	200
152	(c1ccn2cnnc2c1)	33	24
153	(O=C1NC=Nc2ccsc12)	114	46
154	(O=C(Nc1ccccc1)C2CCNCC2)	40	40
155	(c1cc2ncnn2cn1)	167	83
156	(O=C1Nc2ccccc2S(=O)(=O)N1)	58	37

157	(O=C1NC=Nc2sccc12)	203	151
158	(C1CN2CCC1c3ncccc23)	17	16
159	(O=C1NCC2=C(CNC2=O)N1)	13	13
160	(O=C1NC(=O)c2ccccc2N1)	571	501
161	(O=C1C=CC2=C1CC=CN2)	11	11
162	(O=C(CSc1ncc[nH]1)Nc2ccccc2)	89	72
163	(C1CNc2ccccc2C1)	299	264
164	(O=C(CSC1=NC(=O)NC=C1)Nc2ccccc2)	13	13
165	(O=S(=O)(Cc1occc1)c2cccs2)	39	39
166	(C1C=CNc2nnnn12)	24	24
167	(O=C(NCCc1occc1)C(=O)NCCc2ccccc2)	12	12
168	(O=C(NCCc1occc1)C(=O)NCc2ccccc2)	11	11
169	(C(N1CCNCC1)c2occc2)	18	18
170	(C1CC(CCO1)c2cccs2)	24	24
171	(O=C1Nc2nccn2C=C1)	12	12
172	(O=C1NNC=C1NS(=O)(=O)c2ccccc2)	6	6
173	(O=C1NC=COc2ccccc12)	4	4
174	(O=C1CC(=O)Nc2ccccc2N1)	25	25
175	(O=C(NCCc1ccccc1)C(=O)NCCc2cccs2)	12	12
176	(O=C(NCCc1cccs1)C(=O)NCc2ccccc2)	13	13
177	(c1cnc2[nH]ccc2n1)	17	17
178	(C(N1CCNCC1)c2cccnc2)	63	63
179	(O=C(CSc1ccccn1)Nc2ccccc2)	56	19
180	(O=C(CNS(=O)(=O)c1conc1)Nc2ccccc2)	13	13
181	(C(c1occc1)n2ccnc2)	13	13
182	(C1NC=Cc2nncnc2O1)	33	18
183	(O=C1NC=Nc2cc[nH]c12)	175	74
184	(O=C1CCCC2=C1C=CC(=O)N2)	62	42
185	(O=C1NC=CC(N1)c2cn[nH]c2)	13	13
186	(c1cnc2nccn2c1)	89	65
187	(O=C(CSC1=NC=CC(=O)N1)Nc2ccccc2)	49	45
188	(O=C1C2CN3CC1CN(C2)C3c4cc[nH]c4)	14	14
189	(O=C(CNc1ccccc1)NCc2ccccc2)	14	14
190	(C1CC2CNC=CN2C1)	26	26
191	(O=C1CCn2nccc2N1)	86	86
192	(O=C1NN=Cc2c[nH]nc12)	105	105
193	(O=C1CCc2ccccc2N1)	107	107
194	(c1cnc2n[nH]cc2c1)	25	6
195	(O=S1(=O)NC=Cc2ncncc12)	106	75
196	(O=C(CNS(=O)(=O)c1cc[nH]c1)N2CCNCC2)	13	13
197	(c1ccc(cc1)n2cnnc2)	9	9

198	(O=C1CNC(=O)C2CCCCN12)	19	19
199	(C1Cn2cnnc2S1)	37	37
200	(C1Cc2ccsc2CN1)	86	53
201	(C(C1CCNCC1)N2CCCCCC2)	13	13
202	(O=C(NCCc1occc1)c2ccccc2)	13	13
203	(C(C1CCNCC1)N2CCCC2)	13	13
204	(c1cnn2ccnc2c1)	13	13
205	(C(C1CCNCC1)N2CCOCC2)	13	13
206	(O=S(=O)(Cc1cccs1)c2cccs2)	26	26
207	(C(C1CCNCC1)N2CCCCC2)	18	18
208	(C1COc2ccccc2N1)	214	212
209	(O=C(CNS(=O)(=O)c1cc[nH]c1)NCc2ccccc2)	13	13
210	(O=C(CNS(=O)(=O)c1cc[nH]c1)Nc2ccccc2)	31	31
211	(O=S(=O)(N1CCCCC1)c2cc[nH]c2)	99	99
212	(O=C(NCc1ccccc1)c2ccn[nH]2)	13	13
213	(O=C1C=CN=C2SC=NN12)	649	595
214	(O=C1CCCc2ccccc2N1)	93	93
215	(C1CSc2ccccc2N1)	137	99
216	(c1cn2ncsc2n1)	69	69
217	(C1CCc2nncn2CC1)	24	24
218	(c1cn2ccsc2n1)	99	87
219	(O=C1OC2(CCNCC2)C=C1)	25	25
220	(o1cccc1c2oncc2)	12	12
221	(O=C1NC=CC=C1CN2CCCCC2)	25	25
222	(o1nccc1c2cccs2)	36	36
223	(o1nccc1c2ccccc2)	112	110
224	(O=C1NCc2ccccc12)	206	205
225	(O=S(=O)(N1CCNCC1)c2ccccc2)	80	54
226	(C(C1CCCCC1)n2cccc2)	12	12
227	(O=S1(=O)C=CC(=N1)NCc2ccccc2)	12	12
228	(O=C1C=CNc2ncccc12)	75	66
229	(O=C1OC=Cc2[nH]cnc12)	22	22
230	(O=C1NC=CC=C1CN2CCNCC2)	43	43
231	(O=C(COCc1ccon1)Nc2ccccc2)	12	12
232	(O=C(NCc1nnn[nH]1)c2ccccc2)	13	13
233	(O=S(=O)(N1CCCCCC1)c2cn[nH]c2)	20	20
234	(O=S(=O)(N1CCCCC1)c2cn[nH]c2)	35	35
235	(O=C(NCCS(=O)(=O)N1CCNCC1)C2CNC(=O)C2)	11	11
236	(O=C1NCC=C(CN2CCNCC2)N1)	17	17
237	(O=C(NC1=NCCC(=O)N1)c2ccccc2)	13	13
238	(o1ncc2cncnc12)	164	127

239	(C(Oc1ccccc1)c2cocn2)	23	18
240	(C1CNc2ccnn2C1)	13	13
241	(C1Nc2cccnc2OC=C1)	47	38
242	(c1cnc2[nH]ncc2c1)	164	99
243	(c1cnc2[nH]cnc2c1)	388	296
244	(C1NCc2ccccc2O1)	43	23
245	(O=C(N1CCCCC1)c2cn[nH]c2)	45	42
246	(O=C1CCSc2ccccc2N1)	67	62
247	(c1ccn(c1)c2ccn[nH]2)	79	59
248	(C1CCc2cc[nH]c2CC1)	23	18
249	(C(Sc1ncc[nH]1)c2ccccc2)	13	13
250	(C1CCN(C1)c2cccnn2)	23	22
251	(C1CN(CCO1)c2cccnn2)	13	13
252	(C1CCN(CC1)c2cccnn2)	42	20
253	(O=C1Nc2nncn2C=C1)	241	230
254	(c1ccc(cc1)c2cccnn2)	30	30
255	(O=C1CN(Cc2occc2)C(=O)N1)	13	13
256	(O=S(=O)(N1CCCC1)c2cc[nH]c2)	25	25
257	(O=C1CN(Cc2cccs2)C(=O)N1)	13	13
258	(O=C(CC1NC(=O)NC1=O)Nc2ccccc2)	69	68
259	(C1CCN(CC1)c2nncs2)	111	87
260	(C1C=CNc2ccnn12)	39	33
261	(c1ccc(cc1)n2cccn2)	26	21
262	(C1CC(CCN1)c2ccn[nH]2)	129	79
263	(o1ncnc1c2c[nH]nn2)	65	65
264	(O=C1C=CSc2ccccc12)	39	39
265	(O=C1NC=CSc2ccccc12)	202	108
266	(c1cn2ncnc2s1)	220	198
267	(O=S(=O)(NCCc1cscn1)c2ccccc2)	13	13
268	(O=C1Nc2ccccc2C=C1)	244	237
269	(O=C(NCCc1cscn1)C(=O)Nc2ccccc2)	31	31
270	(C1Cc2ccccc2N1)	884	872
271	(O=C1NC(=O)c2[nH]ccc2N1)	158	73
272	(O=C1NC=Nc2occc12)	69	69
273	(o1ccc2cncnc12)	151	151
274	(c1cn2nnnc2cn1)	52	52
275	(O=S(=O)(N1CCOCC1)c2cc[nH]c2)	13	13
276	(O=C(Cc1ccccc1)NCCc2cscn2)	12	12
277	(O=C(NCCc1cscn1)c2ccccc2)	31	27
278	(O=S(=O)(NCCc1cncs1)c2ccccc2)	17	17
279	(O=C(NCCc1cncs1)C(=O)Nc2ccccc2)	29	29

280	(O=C(NCCc1cncs1)c2ccccc2)	31	28
281	(O=C(NCCc1cccs1)c2ccccc2)	13	10
282	(O=S1(=O)CCCCN1c2ccccc2)	42	42
283	(O=C1Nc2ccccc2NC1=O)	130	130
284	(O=C1CC(=O)c2ccccc12)	21	13
285	(O=C(NCc1ccccc1)c2cncnc2)	20	20
286	(O=C(Nc1ccccc1)c2cncnc2)	18	17
287	(O=C1NN=Cc2cc[nH]c12)	353	246
288	(C1CCc2cnoc2C1)	18	18
289	(O=C(NCCc1ccccc1)c2c[nH]nn2)	13	13
290	(O=C(NCc1cccs1)c2c[nH]nn2)	13	13
291	(O=C(NCc1ccccc1)c2c[nH]nn2)	19	19
292	(C(N1CCOCC1)c2ccccc2)	39	39
293	(C(N1CCCC1)c2ccccc2)	16	16
294	(O=C(CN1C=CC=CC1=O)Nc2ccccc2)	13	13
295	(O=C(Nc1ccccc1)c2ccon2)	29	25
296	(o1cncc1c2ccccc2)	45	45
297	(O=C(NCc1ccc[nH]1)Nc2ccccc2)	12	12
298	(O=C(Nc1cnon1)c2occc2)	6	6
299	(O=C(COc1ccccc1)Nc2cnon2)	12	12
300	(O=C(Nc1ccccc1)c2c[nH]nn2)	69	69
301	(c1ncc2nc[nH]c2n1)	32	32
302	(O=S(=O)(N1CCNCC1)c2cc[nH]c2)	17	17
303	(C1CCc2nccn2CC1)	87	87
304	(O=C1NC=Nc2oncc12)	65	65
305	(c1ccc2nccnc2c1)	242	88
306	(O=C1NC=CSc2ncccc12)	24	24
307	(O=C1NC=Cc2ncccc12)	99	99
308	(O=S1(=O)N=Cc2ccccc12)	9	9
309	(O=S(=O)(NCc1oncn1)c2ccccc2)	10	10
310	(O=C(CCn1cccn1)NCc2ccccc2)	11	11
311	(O=C(CCn1cccn1)Nc2ccccc2)	29	29
312	(C(Sc1nnc[nH]1)c2oncn2)	63	27
313	(O=C(CCCc1oncn1)Nc2ccccc2)	13	13
314	(O=S1(=O)C=CNc2ccccc12)	22	19
315	(c1ccc2sccc2c1)	50	46
316	(O=C1CSC2(N1)C=CNC2=O)	102	84
317	(O=C1OC2(CCCCCC2)C=C1)	18	18
318	(O=C1OC2(CCCCC2)C=C1)	35	35
319	(O=C(Cn1cccc1)Nc2ccccc2)	13	13
320	(O=C(CSc1ncccn1)Nc2ccccc2)	10	10

321	(c1cc2nncn2cn1)	37	33
322	(O=C(CNS(=O)(=O)c1ccsc1)Nc2ccccc2)	12	12
323	(O=S(=O)(Nc1ccccc1)c2ccsc2)	28	20
324	(O=S(=O)(N1CCNCC1)c2ccsc2)	24	22
325	(O=C(CNS(=O)(=O)c1cccs1)Nc2ccccc2)	39	37
326	(O=C1Nc2[nH]ncc2C=C1)	176	166
327	(O=C(Nc1nncs1)c2ccccc2)	54	54
328	(O=S1(=O)NCCCN1Cc2ccccc2)	9	9
329	(O=C(CN1CCNS1(=O)=O)Nc2ccccc2)	10	10
330	(c1cn2cnnc2cn1)	64	55
331	(C1Cn2cnnc2C=N1)	23	7
332	(C1CSc2ncccc2N1)	63	60
333	(C1CCc2[nH]ccc2C1)	43	16
334	(C1CCc2ccccc2NC1)	51	35
335	(O=C1NC=Nc2[nH]ncc12)	68	44
336	(O=C(NC1CCCCCC1)C2CNCC(=O)N2)	12	12
337	(O=C1NC=COc2ncccc12)	58	58
338	(c1nncc2n[nH]cc12)	122	86
339	(C(NC1CCNCC1)c2ccccc2)	6	6
340	(C1CCN2CCNCC2C1)	13	13
341	(C(NC1CCNCC1)c2occc2)	13	13
342	(O=S1(=O)C=CC(=N1)N2CCCCC2)	49	49
343	(O=C1NN2C=NC=NC2=C1)	72	50
344	(o1ncc2ccccc12)	61	60
345	(O=C1C=CN=C2CCCCCN12)	77	76
346	(O=C1NC=CN=C1NCCc2ccccc2)	13	13
347	(O=C1NC=CN=C1NCc2ccccc2)	13	13
348	(c1nc(ns1)c2c[nH]nn2)	34	23
349	(O=C(NCc1oncn1)c2ccccc2)	30	27
350	(C1Nc2sccc2C=N1)	13	11
351	(c1csc(c1)c2ccncn2)	13	4
352	(O=C1NC=Cc2sccc12)	90	83
353	(O=C(NCc1ccccc1)C2CCCCC2)	7	7
354	(O=C(NCCc1ccccc1)C2CCCCC2)	11	9
355	(O=C1OC=Cc2sccc12)	65	39
356	(O=S1(=O)NC=Cc2ccccc12)	56	53
357	(N1C=CS/C/1=N\c2ccccc2)	5	5
358	(O=C1CCCc2ncncc12)	6	6
359	(c1ccc(cc1)c2cc[nH]n2)	14	10
360	(O=S1(=O)CCC(C1)NCc2cccs2)	23	23
361	(o1cccc1c2ccccc2)	11	4

362	(O=C1C=CNc2ncnn12)	19	18
363	(C(CSc1nnn[nH]1)NCc2ccccc2)	36	36
364	(O=C1CCC2=C(N1)NC(=O)NC2=O)	6	6
365	(C1CCNCC1)	13	13
366	(c1c[nH]c(c1)c2cccs2)	13	7
367	(O=C(CSc1nnc[nH]1)Nc2ccccc2)	42	42
368	(C1Cc2ncccc2CN1)	26	26
369	(O=S(=O)(Nc1ccccc1)c2cccs2)	13	13
370	(O=C1NN=Nc2ccsc12)	13	5
371	(O=C(NCc1ccccc1)C2CCNCC2)	16	16
372	(c1cnc2nncn2c1)	48	47
373	(O=S(=O)(N1CCCCC1)c2conc2)	120	108
374	(O=C(Nc1cccs1)\C=C\c2ccccc2)	11	8
375	(O=C1C=COc2ccccc12)	204	170
376	(C(=C\c1nccs1)/c2ccccc2)	10	1
377	(O=C(COc1ccccc1)Nc2nnc[nH]2)	6	6
378	(N=C1Nc2ccccc2N1)	5	5
379	(O=C1NC(=O)C2=CC=CNC2=N1)	23	23
380	(O=C1OCCc2ccccc12)	27	19
381	(c1nncc2c[nH]cc12)	1	1
382	(O=S(=O)(N1CCCC1)c2ccccc2)	12	12
383	(C(Sc1ocnn1)c2ccccc2)	18	18
384	(O=C(NCC1CCCO1)\C=C\c2ccccc2)	6	6
385	(O=C(CSc1nnc[nH]1)N2CCCCC2)	12	12
386	(O=C1NCC=C(CN2CCCCC2)N1)	6	6
387	(C(Oc1ccccc1)c2ocnn2)	17	17
388	(C(Cc1ccccc1)Cc2ocnn2)	11	11
389	(N(c1ccccc1)c2ncccn2)	2	2
390	(O=C(CSc1nccnn1)Nc2ccccc2)	22	12
391	(c1ncc2ccsc2n1)	63	22
392	(O=C(Nc1ccccc1)c2conc2)	11	11
393	(O=C(CCn1cccc1)NC2CCCCC2)	8	8
394	(O=C(NS(=O)(=O)c1ccccc1)c2ccccc2)	13	5
395	(O=C(COc1ccccc1)NCc2oncn2)	13	13
396	(O=C(Nc1ncns1)c2ccccc2)	13	13
397	(c1ccc(cc1)n2cccc2)	9	0
398	(c1cc(ccn1)c2nnc[nH]2)	7	7
399	(O=C(CCC(=O)c1ccccc1)Nc2ccccc2)	11	11
400	(O=S(=O)(N1CCNCC1)c2cccs2)	17	15
401	(O=C1NC=C(C(=O)N1)S(=O)(=O)N2CCNCC2)	5	5
402	(O=C(Cn1cccc1)NC2CCCCC2)	5	5

403	(O=C(Cn1cccc1)NC2CCCCCC2)	5	3
404	(O=S(=O)(NCC1CCCCC1)c2cccs2)	12	12
405	(O=C(C1CCCCC1)N2CCNCC2)	8	8
406	(O=S(=O)(N1CCCCC1)c2cccs2)	20	20
407	(C1C=CNc2nccn12)	31	13
408	(C1CCc2ccsc2CC1)	92	35
409	(O=C(CS(=O)Cc1cocn1)N2CCNCC2)	9	9
410	(O=C(NC1CCCCC1)c2occc2)	6	5
411	(O=C(CN1CCNC1=O)NCc2ccccc2)	12	12
412	(O=C(NCc1ccccc1)c2ccccc2)	22	21
413	(O=C(NCCc1ccccc1)c2ccccc2)	5	5
414	(C(SCc1ccccc1)c2occc2)	23	0
415	(C1Cc2ccsc2C1)	62	56
416	(O=C1Nc2ccccc2SC=C1)	5	0
417	(O=C1Nc2ccccc2S(=O)C=C1)	5	5
418	(S1C=CC=Nc2ccccc12)	28	8
419	(O=C(C1CCNCC1)N2CCNCC2)	62	57
420	(C1CC(CN1)c2ccccc2)	8	8
421	(O=C(CS(=O)Cc1cocn1)NCc2ccccc2)	12	12
422	(O=C(CCSCCc1ccccn1)Nc2ccccc2)	7	7
423	(O=C(CSCc1cocn1)NCCc2ccccc2)	12	12
424	(O=C(CCSCc1ccccc1)NCc2ccccc2)	11	11
425	(O=C(CCSCc1ccccc1)NCCc2ccccc2)	10	10
426	(O=S(=O)(N1CCCCC1)N2CCCCC2)	8	8
427	(O=C(NCCCNc1ccccc1)c2ccccc2)	5	1
428	(O=C(NCCc1ccccc1)C2CNC(=O)C2)	6	6
429	(O=S(=O)(N1CCCCC1)N2CCNCC2)	5	5
430	(O=C1CN(C2CCCCCC2)C(=O)CN1)	37	27
431	(C(NCc1ccccc1)C2CCCCC2)	11	1
432	(O=C(CSc1nccnn1)NCc2ccccc2)	8	0
433	(O=C(CSc1nccnn1)NC2CCCCC2)	7	0
434	(O=C(CSc1nccnn1)NCCc2ccccc2)	7	0
435	(c1ccc(cc1)c2cncnn2)	20	3
436	(O=C(CNC(=O)\C=C/C(=O)Nc1ccccc1)NCc2occc2)	6	0
437	(c1ncc2sccc2n1)	14	1
438	(N=C1NC=Nc2n[nH]cc12)	1	1
439	(C(Sc1ccccn1)c2ccccc2)	5	3
440	(O=S(=O)(NCCC1=CCCCC1)c2ccccc2)	5	3
441	(O=S(=O)(NC1CCCCCC1)c2ccccc2)	8	8
442	(O=C(Oc1ccccc1)c2conc2)	8	4
443	(o1ccc2[nH]ccc12)	73	46

444	(c1cc2sccc2[nH]1)	111	70
445	(O=S(=O)(NC1CCCCC1)c2ccccc2)	14	14
446	(c1ccc2ccccc2c1)	42	25
447	(C1Cc2cc[nH]c2CN1)	49	3
448	(O=S1(=O)Cc2c[nH]nc2C1)	14	14
449	(O=C1C=CN=C2SCC=NN12)	9	0
450	(O=C1CN=C2C=CN=CN12)	191	129
451	(O=S1(=O)N=CNc2ccccc12)	17	17
452	(O=S1(=O)NC=Nc2ccccc12)	11	11
453	(O=C(c1ccccc1)c2cccs2)	29	0
454	(N(c1ccccc1)c2cccs2)	33	12
455	(O=C1Nc2cnnn2C=C1)	12	12
456	(O=C(Nc1ccccn1)C2CCCNC2)	8	8
457	(O=C(C1CCCNC1)N2CCNCC2)	22	18
458	(c1cn2nc[nH]c2n1)	11	11
459	(o1ccc2ncncc12)	11	11
460	(O=C1CNC(=O)c2ccccc2N1)	7	7
461	(O=C(Cc1ccc[nH]1)Nc2ccccc2)	12	12
462	(O=C(Nc1ccccn1)C2CCNCC2)	6	6
463	(O=C(Cc1ccc[nH]1)NCc2ccccc2)	9	9
464	(O=C(Cc1ccc[nH]1)N2CCNCC2)	11	11
465	(O=C1Nc2ccccc2N=C1)	66	64
466	(C1CC(CCN1)c2ccncc2)	17	17
467	(O=C1NCCN(C2CCNCC2)C1=O)	47	47
468	(O=C1NN=C(N=C1)c2ccccc2)	5	5
469	(C1CN(CCN1)c2ccccn2)	3	3
470	(C1CN(CC=C1)c2ccccn2)	1	0
471	(C1CCN(CC1)C2CCNCC2)	14	14
472	(O=C(CN1CCNCC1)c2ccccc2)	3	3
473	(C1CC(=CCN1)c2ccccc2)	1	0
474	(C(CN1CCOCC1)C2CCNCC2)	1	1
475	(O=C1C=CNC(=C1)CN2CCCCC2)	9	9
476	(C1CN(CCO1)c2ccccc2)	2	2
477	(O=C(Nc1ccccc1)C2=NNC(=O)C=C2)	7	7
478	(C(Nc1ccccc1)c2ccccc2)	20	17
479	(C1CCN(CC1)c2ccccc2)	1	1
480	(O=C(CNc1ccccc1)NCCc2ccccc2)	8	8
481	(O=C(CSc1nnc[nH]1)c2ccccc2)	20	11
482	(O=C1NC=Cc2ncnn12)	44	36
483	(O=C(Nc1ccccc1)C2=CC=CNC2=O)	28	28
484	(O=C(Nc1nncs1)C2=CC=CNC2=O)	10	10

485	(O=C(Nc1ccon1)C2=CC=CNC2=O)	8	8
486	(O=S(=O)(Cc1cccs1)c2ccccc2)	7	7
487	(C1Cn2cccc2CN1)	18	12
488	(O=S(=O)(Cc1occc1)c2ccccc2)	9	9
489	(O=C1NC2=C(CCC2)C=N1)	82	82
490	(N1C=CSc2nncn12)	30	30
491	(C1SCc2n[nH]cc12)	23	23
492	(O=C(Nc1ccccc1)C2=CNC(=O)C=C2)	9	9
493	(C1CCc2ccccc2C1)	11	5
494	(O=C1NC(=NC=C1)n2cccn2)	9	9
495	(O=C1NC2=C(CCCC2)C=N1)	19	19
496	(O=C(CSC1=NC(=O)NC=C1)NCc2ccccc2)	8	8
497	(C1CN(CCN1)c2cccnn2)	7	7
498	(O=C1NC(=O)c2nc[nH]c2N1)	6	6
499	(O=C(NC1CNC(=O)C1)c2cccs2)	10	10
500	(O=C(NC1CNC(=O)C1)c2occc2)	12	12
501	(O=C(NC1CNC(=O)C1)c2ccccc2)	21	21
502	(O=C(CCc1ccccc1)NC2CNC(=O)C2)	12	12
503	(O=C(Cc1cccs1)NC2CNC(=O)C2)	10	10
504	(O=C(Cc1ccccc1)NC2CNC(=O)C2)	18	18
505	(O=C(COc1ccccc1)NC2CNC(=O)C2)	8	8
506	(O=C(Nc1ccccc1)Nc2nncs2)	10	8
507	(O=C(NC1CNC(=O)C1)\C=C/c2ccccc2)	8	8
508	(O=C(NC1CNC(=O)C1)C2CCCCC2)	10	10
509	(O=C(CSc1nncs1)N2CCCCC2)	4	4
510	(O=C1CCCN1c2ccccc2)	17	17
511	(O=C(NC1CCCCC1)Nc2nncs2)	6	6
512	(O=C(CCCc1ccccc1)NC2CNC(=O)C2)	6	6
513	(c1ncc2nn[nH]c2n1)	73	66
514	(C(N1CCOCC1)c2occc2)	9	9
515	(O=C(COc1ccccc1)NCCc2occc2)	5	5
516	(O=C(CSC1=NC=CC(=O)N1)Nc2ccon2)	5	5
517	(O=C(CSC1=NC=CC(=O)N1)N2CCOCC2)	6	6
518	(O=C(Cc1ccccc1)NCCc2cccs2)	9	8
519	(O=C(COc1ccccc1)NCCc2cccs2)	5	5
520	(O=C(CCc1ccccc1)NCCc2cscn2)	6	6
521	(c1cncc(c1)c2nccs2)	10	10
522	(c1cnnc(c1)c2cncs2)	31	19
523	(C(c1ccccc1)n2ccnc2)	5	5
524	(O=C(CSc1ncc[nH]1)Nc2nncs2)	13	13
525	(O=C1NC(=O)C=C(N1)N2CCNCC2)	5	5

526	(O=C(CSc1ncc[nH]1)NCc2occc2)	5	5
527	(O=C(CSc1ncc[nH]1)NCc2ccccc2)	11	11
528	(O=C(COc1ccccc1)NCCc2cscn2)	7	7
529	(O=C(NCCc1cncs1)c2occc2)	5	5
530	(O=C(Cc1ccccc1)NCCc2cncs2)	7	7
531	(O=C(NCCc1cncs1)c2cccs2)	5	5
532	(O=C(NCCc1cscn1)c2occc2)	8	8
533	(C1CCC(NC1)c2cccnc2)	5	5
534	(c1cn2cnnc2s1)	16	14
535	(O=S(=O)(c1ccccc1)c2cocn2)	2	2
536	(C1CN(CCO1)c2ocnc2)	5	5
537	(C1CCN(CC1)c2ocnc2)	39	30
538	(O=C(Nc1ccccc1)N2CCCC2)	7	7
539	(O=S(=O)(NCc1ccccc1)c2cccs2)	7	7
540	(O=C(CNS(=O)(=O)c1cccs1)N2CCNCC2)	8	8
541	(O=C(CNS(=O)(=O)c1cccs1)NCc2ccccc2)	7	7
542	(o1cnc(c1)c2ccccc2)	21	21
543	(O=C1NN=C(C=C1)c2ccncc2)	5	5
544	(C1CCCc2[nH]ccc2CC1)	8	4
545	(O=C1NN=C2CCCCC2=C1)	16	16
546	(c1ccc2cnccc2c1)	75	59
547	(O=S(=O)(NCc1ccccc1)c2cncnc2)	12	12
548	(O=S(=O)(Nc1ccccc1)c2cncnc2)	8	8
549	(O=S(=O)(N1CCNCC1)c2cncnc2)	2	2
550	(o1ccc2cccnc12)	27	24
551	(C(Sc1ncccn1)c2oncn2)	20	11
552	(c1ccc2sncc2c1)	92	88
553	(O=C(CCc1cc[nH]c1)NCc2ccccc2)	12	12
554	(O=C(CCc1cc[nH]c1)Nc2ccccc2)	11	11
555	(O=C(CCc1cc[nH]c1)N2CCNCC2)	7	7
556	(C1CN(CCN1)c2oncc2)	8	6
557	(O=C(NCc1occc1)c2conc2)	5	5
558	(O=C1C=CN=C2SC=CN12)	311	311
559	(C1CC(=CCN1)c2nocn2)	5	5
560	(O=C(COc1ccccc1)Nc2ccn[nH]2)	12	1
561	(O=C(Nc1ccn[nH]1)c2ccccc2)	11	2
562	(O=C1NC(=O)c2cncnc2N1)	45	45
563	(c1cnc(nc1)n2cccn2)	16	9
564	(C1CCN(CC1)c2ccn[nH]2)	14	14
565	(C1CCN(CC1)c2oncn2)	13	11
566	(C1COc2ccccc2C1)	60	60

567	(C1CC2(CCN1)NC=CN=C2)	7	6
568	(O=C(CSc1nnc[nH]1)N2CCNCC2)	9	9
569	(O=C(CSc1nnc[nH]1)NCc2ccccc2)	9	9
570	(O=C1N=CNc2ncccc12)	13	9
571	(C1CCN2CCN=C2CC1)	26	26
572	(C1CCC2=NCCN2C1)	17	17
573	(O=C(CSc1nnc[nH]1)NCCCc2ccccc2)	7	7
574	(O=C(NCc1nnn[nH]1)C2CNC(=O)C2)	5	5
575	(O=C(CSc1nnc[nH]1)NCCc2ccccc2)	7	7
576	(O=C1NN=Cc2n[nH]cc12)	24	23
577	(O=C(CCc1ccc[nH]1)NCc2ccccc2)	10	10
578	(O=C(CCc1ccc[nH]1)NCCCN2CCNCC2)	3	3
579	(O=C(CCc1ccc[nH]1)Nc2ccccc2)	9	9
580	(C1CN2CCN=C2CO1)	21	21
581	(O=S(=O)(N1CCNCC1)c2cc[nH]n2)	12	12
582	(O=S(=O)(Nc1ccccc1)c2cc[nH]n2)	11	11
583	(O=C(Cc1ccccc1)NCCS(=O)(=O)N2CCNCC2)	5	5
584	(o1cnnc1c2c[nH]nn2)	5	5
585	(C1C=Nc2ccccc2N=C1)	24	9
586	(O=S(=O)(N1CCOCC1)c2cn[nH]c2)	12	12
587	(O=S(=O)(NCc1ccccc1)c2cc[nH]n2)	4	4
588	(O=C(Nc1ccccc1)C2=NNC(=O)NC2=O)	12	12
589	(O=C(N1CCNCC1)C2=NNC(=O)NC2=O)	7	7
590	(O=C(N1CCCCC1)C2=NNC(=O)NC2=O)	5	5
591	(O=C1NC(=O)N(N=C1)c2ccccc2)	8	8
592	(O=C1Nc2ccccc2S1)	27	24
593	(O=C1NC=CN2CCCCC12)	28	28
594	(C1Oc2ccccc2C=C1)	10	10
595	(O=C1NC=Nc2ncccc12)	28	28
596	(O=C1Nc2cncnc2N1)	44	44
597	(O=C(N1CCNCC1)c2ccc[nH]2)	18	18
598	(O=S(=O)(Nc1ccccc1)c2c[nH]cn2)	6	6
599	(O=S(=O)(N1CCNCC1)c2c[nH]cn2)	7	7
600	(C1CCCN(CC1)c2cccnn2)	21	13
601	(O=S(=O)(N1CCCCCC1)c2cc[nH]c2)	27	27
602	(C1CCCc2scnc2CC1)	5	4
603	(C1CN=C2SC=CC2=C1)	5	4
604	(O=C(NCCc1cscn1)c2cccs2)	3	3
605	(O=C(CSC1=NC=CC(=O)N1)NCc2ccccc2)	9	9
606	(O=C1NCCCn2nccc12)	29	29
607	(C1SC=Cc2[nH]ncc12)	42	39

608	(o1cccc1c2ocnc2)	12	12
609	(O=C1Nc2ccccc2C=N1)	61	42
610	(C1CCN(C1)c2ccccc2)	33	25
611	(O=C(NCCc1cn[nH]c1)Nc2ccccc2)	29	29
612	(O=S(=O)(NCc1ccc[nH]1)c2ccccc2)	12	12
613	(O=C1Nc2[nH]ncc2N=C1)	4	4
614	(C1Cc2ncncc2CN1)	78	75
615	(O=C1NC=Cn2nccc12)	22	22
616	(C1CCN(C1)c2ncccn2)	19	19
617	(O=C(N1CCNCC1)c2cncnc2)	14	14
618	(O=C1Oc2cnccc2C=C1)	11	11
619	(O=S(=O)(N1CCCCC1)c2c[nH]cn2)	43	43
620	(O=C(CCn1cccn1)N2CCNCC2)	5	5
621	(O=C1NCCN(C2CCCCC2)C1=O)	7	7
622	(O=C(N1CCOCC1)c2cccs2)	6	6
623	(O=C1NCCN(Cc2ccccc2)C1=O)	4	4
624	(c1ccc2[nH]nnc2c1)	46	44
625	(O=C(NCc1cocn1)NC2CCCCC2)	7	7
626	(O=C(NCc1cocn1)Nc2ccccc2)	7	7
627	(O=C(N1CCCCC1)c2cc[nH]c2)	21	21
628	(O=S(=O)(NCc1cocn1)c2ccccc2)	4	4
629	(O=C(CN1CCCNS1(=O)=O)Nc2ccccc2)	27	27
630	(O=C(CN1CCCNS1(=O)=O)NCc2ccccc2)	4	4
631	(c1cc2[nH]ncc2cn1)	79	19
632	(O=C1CC=Nc2ccccc2N1)	17	15
633	(O=C1NC(=O)c2sccc2N1)	255	192
634	(C1CN2C=NC=CC2=N1)	12	12
635	(C(N1CCCC1)c2oncc2)	12	12
636	(O=C1CSc2[nH]ncc2N1)	27	27
637	(C(N1CCOCC1)c2oncc2)	12	12
638	(O=S(=O)(N1CCCC1)c2c[nH]cn2)	8	8
639	(c1cnnc(c1)n2cccn2)	70	68
640	(O=C(CCc1cn[nH]c1)N2CCNCC2)	6	5
641	(O=C1CC(CN1)c2oncn2)	10	10
642	(O=C(N1CCCCCC1)c2cc[nH]c2)	17	17
643	(O=C(CNS(=O)(=O)c1ccsc1)NCc2ccccc2)	10	10
644	(C(Nc1ccccc1)c2ocnn2)	29	3
645	(C(N1CCNCC1)c2ocnn2)	29	17
646	(O=C1C=CS(=O)(=O)N1Cc2ccccc2)	23	21
647	(O=C1NCc2cccnc12)	45	40
648	(o1ncnc1c2ccn[nH]2)	22	22

649	(O=S(=O)(c1ccccc1)c2cn[nH]c2)	23	23
650	(c1ccn2cccc2c1)	33	25
651	(O=C1NC=CN=C1NCc2cccs2)	5	5
652	(O=C1NC=CN2CCCC12)	8	8
653	(O=S(=O)(NCC1CCNCC1)c2cc[nH]c2)	8	8
654	(O=S1(=O)CCCN1c2ccccc2)	19	19
655	(o1ccc(n1)c2cccs2)	9	9
656	(O=C1NC=Nc2c[nH]nc12)	30	8
657	(c1ccn(c1)c2nncs2)	15	9
658	(C1CN(CCN1)c2nncs2)	4	4
659	(C1CCN(C1)c2nncs2)	18	18
660	(O=S(=O)(NCC1CCCCC1)c2ccsc2)	6	6
661	(O=S(=O)(NCc1ccccc1)c2ccsc2)	11	11
662	(C1CCC2=C(CC1)NCC2)	22	15
663	(O=C(N1CCNCC1)c2cn[nH]c2)	86	53
664	(O=C(NC1CCNCC1)c2cn[nH]c2)	6	6
665	(O=S(=O)(N1CCCCC1)c2ccsc2)	6	6
666	(O=C1CNc2ccccc12)	12	12
667	(c1ccc2nnccc2c1)	10	6
668	(c1ccn2nccc2c1)	29	29
669	(O=C1NN=C(C=C1)N2CCNCC2)	42	42
670	(O=C(CSc1oncn1)N2CCNCC2)	8	8
671	(O=C(CSc1oncn1)Nc2ccccc2)	9	8
672	(O=C(CSc1oncn1)N2CCCCC2)	6	6
673	(O=C(NC1=CNC(=O)NC1=O)Nc2ccccc2)	12	12
674	(O=C(CNS(=O)(=O)c1cn[nH]c1)Nc2ccccc2)	10	10
675	(O=C(NCc1ccccc1)C2CCCCN2)	12	12
676	(C1CC2(CCN1)N=CC=N2)	11	2
677	(C1Nc2ccccc2C=N1)	5	5
678	(O=C(NCCCN1CCNCC1)c2cn[nH]c2)	23	19
679	(O=C1CS(=O)(=O)C2(N1)C=CNC2=O)	40	40
680	(C1CN(CCN1)c2ncccn2)	8	8
681	(O=C(NCCN1CCNCC1)c2cn[nH]c2)	8	8
682	(O=C(N1CCOCC1)c2cn[nH]c2)	5	5
683	(O=C(NCCN1CCOCC1)c2cn[nH]c2)	8	8
684	(O=C1CCSC2(N1)C=CNC2=O)	9	8
685	(C1CCCN(CC1)c2nncs2)	14	12
686	(C1CN(CCO1)c2nncs2)	5	5
687	(C1CCc2oncc2CC1)	9	9
688	(O=C(NCc1occc1)c2c[nH]nn2)	7	7
689	(O=C(Cn1cccc1)N2CCNCC2)	8	8

690	(O=S(=O)(NCc1ccccc1)c2nncs2)	8	8
691	(O=C1NCc2cc[nH]c12)	16	10
692	(C(N1CCNCC1)c2nocn2)	7	7
693	(O=C(NCCc1ccccc1)c2ccn[nH]2)	8	8
694	(O=C(Nc1ccccc1)c2ccn[nH]2)	10	10
695	(O=C1NN=C2C=CNC=C12)	11	11
696	(O=S(=O)(Cc1cocn1)c2ccccc2)	10	10
697	(C(Sc1ccccc1)c2cocn2)	29	10
698	(O=C(N1CCNCC1)c2ccccc2)	7	1
699	(O=C1CN(CCc2ccncc2)C(=O)N1)	7	7
700	(O=C1CN(Cc2cccnc2)C(=O)N1)	9	9
701	(O=C(CSCc1oncn1)Nc2ccccc2)	5	5
702	(C1CN(CCN1)c2oncn2)	10	10
703	(C1CCc2cncn2CC1)	30	11
704	(c1cnc2cnnn2c1)	225	128
705	(O=C(NC1CCCCC1)C2CNCC(=O)N2)	11	11
706	(O=C1CNCCN1c2ccccc2)	4	4
707	(O=C(NCc1ccccc1)c2ccccn2)	1	1
708	(O=C(N1CCNCC1)c2ccccn2)	1	1
709	(O=C(N1CCNCC1)c2ccn[nH]2)	13	13
710	(c1cc2ccsc2[nH]1)	11	1
711	(O=C(COCc1ccon1)N2CCNCC2)	3	3
712	(O=C1NC=CC(N1)c2ccccc2)	19	19
713	(c1cc2cn[nH]c2s1)	62	38
714	(C1SC=CN=C2C=CC=C12)	8	2
715	(O=C1CCC=C2SCNCN12)	8	7
716	(C1CCc2ncccc2C1)	39	6
717	(O=C1NC=Cc2[nH]ccc12)	24	24
718	(O=S(=O)(Nc1ccccc1)c2conc2)	5	5
719	(O=C1OC=Cc2[nH]ncc12)	39	39
720	(O=C(CNS(=O)(=O)c1cn[nH]c1)NCc2ccccc2)	12	12
721	(O=C(CNS(=O)(=O)c1conc1)N2CCNCC2)	12	12
722	(O=C(CNS(=O)(=O)c1conc1)NCc2ccccc2)	12	12
723	(O=C(CNS(=O)(=O)c1conc1)NCCc2ccccc2)	7	7
724	(O=C(Cc1oncn1)Nc2ccccc2)	10	10
725	(c1ccc(nc1)c2cccnn2)	3	3
726	(c1cnnc(c1)c2cccs2)	17	13
727	(O=C1NC(=O)c2occc2N1)	83	65
728	(C1C=COc2ncncc12)	98	25
729	(O=C(NCCCN1CCNCC1)c2occc2)	1	1
730	(O=C(N1CCNCC1)c2occc2)	13	10

731	(O=C(NC1CCCCCC1)c2occc2)	5	5
732	(C(N1CCNCC1)c2ccsc2)	15	8
733	(O=C1Nc2ncccc2N=C1)	110	110
734	(O=C1Nc2cccnc2SC=C1)	30	30
735	(c1cc2cnncn2c1)	86	63
736	(O=C(NCCc1ccccc1)C2CCCNC2)	5	5
737	(O=C1COc2cccnc2N1)	11	11
738	(O=C(NCc1ccccc1)C2CCCNC2)	12	12
739	(O=C(Nc1ccccc1)C2CCCNC2)	11	11
740	(O=C1CCOc2ccccc12)	18	17
741	(C1Cc2c[nH]nc2C=C1)	63	37
742	(O=C(NCC12CC3CC(CC(C3)C1)C2)c4ccccc4)	4	0
743	(C1Cn2ccnc2S1)	101	55
744	(O=C1NC=Nc2ccoc12)	77	47
745	(O=C(Nc1ccccc1)c2oncc2)	8	8
746	(C1OC=Cc2oncc12)	13	12
747	(O=C1NC=Cn2cccc12)	103	103
748	(c1cc2cnccn2c1)	6	5
749	(O=C1C=CN=C2CCCN12)	29	29
750	(O=C1NC=CN(Cc2ccccc2)C1=O)	9	9
751	(O=C(CN1C=CNC(=O)C1=O)Nc2ccccc2)	39	39
752	(C(c1ccccc1)c2ocnn2)	15	15
753	(O=C1NC=CN(C1=O)c2ccccc2)	12	12
754	(O=C(NCCCN1CCNCC1)c2ocnn2)	6	6
755	(O=C(CN1C=CNC(=O)C1=O)NCc2ccccc2)	8	8
756	(O=C(CN1C=CNC(=O)C1=O)c2ccccc2)	5	5
757	(O=C(CN1C=CNC(=O)C1=O)NCCc2ccccc2)	6	6
758	(O=S1(=O)Cc2cn[nH]c2C=C1)	40	40
759	(O=C1C=CN=C2CCCCN12)	27	27
760	(O=C(NCc1ccccc1)c2cc[nH]c2)	7	7
761	(O=C(NCCc1ccccc1)c2cc[nH]c2)	5	5
762	(O=C1NC=Nc2nccnc12)	17	14
763	(O=C(Nc1nncs1)C2CCCCC2)	12	12
764	(O=S(=O)(Nc1ccccc1)c2nncs2)	11	11
765	(O=S(=O)(N1CCCCCC1)c2nncs2)	24	24
766	(O=C(COc1ccccc1)Nc2nncs2)	19	19
767	(O=S(=O)(N1CCOCC1)c2nncs2)	8	8
768	(O=C(Nc1nncs1)c2occc2)	12	12
769	(O=S(=O)(NCc1occc1)c2nncs2)	6	6
770	(O=S(=O)(N1CCCCC1)c2nncs2)	9	9
771	(C(N1CCOCC1)c2cccnc2)	9	9

772	(O=C(Nc1nncs1)c2cccs2)	8	8
773	(O=S(=O)(N1CCNCC1)c2nncs2)	12	12
774	(O=S(=O)(N1CCCC1)c2nncs2)	10	10
775	(c1cnc2scnc2c1)	31	29
776	(O=C(Nc1ccccc1)C(=O)c2cc[nH]c2)	12	12
777	(O=C(Nc1nncs1)C(=O)c2cc[nH]c2)	11	11
778	(O=C(Nc1ccccc1)c2cn[nH]c2)	12	4
779	(O=C1NN=Cn2cccc12)	270	261
780	(O=S1(=O)NC=CC(=N1)c2cccs2)	10	10
781	(O=S1(=O)NC=CC(=N1)c2occc2)	7	7
782	(O=C1NN=Cc2sccc12)	69	66
783	(O=C1NC=NC2=C1CNCC2)	25	25
784	(O=C(Nc1ccccc1)C2=CC=NS(=O)(=O)N2)	12	12
785	(O=C(NCc1ccccc1)C2=CC=NS(=O)(=O)N2)	5	5
786	(C1OC=Cc2ncncc12)	9	0
787	(C1OC=Cc2[nH]ncc12)	34	34
788	(C1SC=Cc2oncc12)	8	8
789	(c1ccc2[nH]ncc2c1)	17	17
790	(O=S1(=O)C=CC(=N1)N2CCNCC2)	12	12
791	(O=S1(=O)C=CC(=N1)NCCCN2CCNCC2)	7	7
792	(N(c1ccccc1)c2nncs2)	9	9
793	(O=C1CNCc2ccsc2N1)	5	3
794	(C1Cc2cn[nH]c2C=C1)	12	12
795	(C1CCC(CC1)Nc2nncs2)	12	12
796	(O=C1NC2(CCNCC2)OC=C1)	12	5
797	(O=C1NN=Cn2nccc12)	10	10
798	(c1cc2[nH]ccn2n1)	20	20
799	(O=C(NCc1cn[nH]c1)Nc2ccccc2)	6	0
800	(C(Nc1nncs1)c2ccccc2)	11	11
801	(o1ncnc1c2ccccc2)	142	138
802	(O=C1CCc2cn[nH]c2N1)	28	24
803	(C1NC=Cn2cccc12)	16	10
804	(O=C(Nc1ccccc1)Nc2cccs2)	8	8
805	(O=C(CSc1ocnn1)Oc2ccccc2)	15	15
806	(O=C1C=CNC2=C1CCCC2)	7	7
807	(O=C(Nc1cnon1)c2ccccc2)	12	12
808	(O=C1NC=NC2=C1CCC2)	17	17
809	(O=C(NCCSCc1ccccc1)C2CCNCC2)	6	6
810	(o1cnnc1c2cccs2)	24	24
811	(O=C1NC=NC2=C1CCCC2)	17	16
812	(O=C(NCc1occc1)c2ccon2)	6	1

813	(C1CNc2nccn2C1)	7	5
814	(C1CC=Nc2ccccc2S1)	5	0
815	(O=C(Nc1cccs1)c2ccon2)	26	18
816	(O=C1CCNC(C1)c2cccs2)	5	5
817	(O=C(N1CCNCC1)c2ccon2)	12	12
818	(O=C1CSCc2cn[nH]c2N1)	11	11
819	(O=C(CSc1ocnn1)NCc2ccccc2)	6	6
820	(C(CSc1ocnn1)Oc2ccccc2)	10	10
821	(O=C(CSc1ocnn1)NC2CCCCC2)	8	8
822	(O=C(Nc1ccccc1)c2nc[nH]n2)	24	24
823	(o1cnc(n1)c2cccnc2)	18	8
824	(O=C(CCc1oncn1)Nc2ccccc2)	29	29
825	(o1ncnc1c2cccnc2)	17	11
826	(o1cnc(n1)c2ccccn2)	14	14
827	(C1CCc2scnc2C1)	35	35
828	(O=C1NC(=O)c2cc[nH]c2N1)	29	29
829	(C(Cc1ccncc1)N2CCNCC2)	23	23
830	(O=C(COc1ccccc1)Nc2ccccc2)	15	2
831	(C(Nc1ccccn1)c2ccccc2)	31	30
832	(O=C(NCc1cccs1)c2ccccc2)	11	4
833	(O=C(NCc1cccs1)c2occc2)	5	5
834	(O=C(COc1ccccc1)NCc2cccs2)	12	6
835	(C(Nc1ccccn1)c2occc2)	23	23
836	(C(Cc1ccccn1)N2CCNCC2)	20	20
837	(O=C(Oc1ccccc1)c2ccon2)	12	5
838	(C1CCC(CC1)N2CCOCC2)	21	21
839	(O=C(NCc1ccccc1)c2ccon2)	17	17
840	(O=C(COc1ccccc1)NCC2CCCCC2)	10	10
841	(C1CCCCC1)	9	7
842	(O=C(NCC1CCCCC1)c2ccccc2)	10	10
843	(C1CC(CCN1)c2ocnn2)	24	24
844	(C(Oc1ccccc1)c2nccs2)	12	12
845	(o1cccc1c2nc[nH]n2)	43	38
846	(C1CCC(CC1)N2CCNCC2)	3	3
847	(C1CCC2(CC1)CCNC=N2)	37	25
848	(c1cnn(c1)c2nccs2)	30	5
849	(O=C1C=CC=Cc2cocc12)	24	21
850	(O=S1(=O)CCC(C1)NCc2occc2)	22	22
851	(o1cnc(n1)c2cccs2)	5	5
852	(O=C(Oc1ccccc1)c2cnns2)	9	9
853	(c1csc(c1)c2ccsc2)	15	13

854	(C(NCc1ccccc1)C2CCCO2)	22	22
855	(O=C(Nc1ccccc1)c2csnn2)	11	11
856	(O=S(=O)(c1ccccc1)c2c[nH]cn2)	7	6
857	(c1csc(c1)c2ncsn2)	6	6
858	(O=C(COc1ccccc1)Nc2ncns2)	11	11
859	(C(N1CCCCC1)c2ccccc2)	17	16
860	(O=C(CSc1ccncn1)Nc2ccccc2)	12	12
861	(o1cccc1c2occn2)	56	33
862	(C(Nc1ocnc1)c2cccnc2)	5	5
863	(C1CN(CCN1)c2ocnc2)	42	25
864	(C1Cc2cncnc2C1)	1	1
865	(O=C(COc1ccccc1)Nc2oncc2)	12	12
866	(O=C1CC(CN1)c2nncs2)	7	7
867	(N(c1ccccc1)c2ccncn2)	37	28
868	(O=C1NC=NC(=C1)c2ccccc2)	1	1
869	(C(CNCc1occc1)CSc2nnn[nH]2)	5	5
870	(C(CNCc1ccccc1)CSc2nnn[nH]2)	23	23
871	(C(Nc1cncnc1)c2occc2)	41	24
872	(o1ncc(n1)c2nnc[nH]2)	36	36
873	(O=C(CSc1ccccn1)c2ccccc2)	5	1
874	(C(CSc1nnn[nH]1)NCc2occc2)	6	5
875	(O=C1CCC2=C(NC=NC2=O)N1)	11	11
876	(C1CCN(CC1)c2cnccn2)	21	21
877	(O=C1CCC2=C(N1)N=CNC2=O)	7	7
878	(O=C(Nc1oncc1)c2ccccc2)	11	11
879	(O=C1NN=Nc2ccccc12)	12	12
880	(O=C(NCC1CCNC1)Nc2ccccc2)	2	2
881	(O=C(NCCN1CCNCC1)Nc2ccccc2)	6	6
882	(O=C(NCc1oncn1)c2occc2)	9	9
883	(o1nc2nccnc2n1)	2	1
884	(O=C(NCC1CCNCC1)Nc2ccccc2)	6	5
885	(C1CCN(CC1)c2ncccn2)	8	8
886	(O=C(Nc1ccccc1)C2=CC=NNC2=O)	12	12
887	(O=C(Nc1nccs1)C2=CC=NNC2=O)	5	5
888	(O=C1NN=CC2=C1CCCC2)	12	5
889	(O=C(Cc1cscn1)Nc2ccccc2)	12	12
890	(O=C(Nc1ccccc1)c2ccncn2)	33	33
891	(O=C(Nc1nncs1)c2ccncn2)	14	14
892	(C(Sc1ncccn1)c2ccccc2)	38	15
893	(C(NCc1ccccc1)c2occc2)	41	22
894	(C(NCc1occc1)c2occc2)	24	24

895	(O=C(NC1CCS(=O)(=O)C1)c2ccncn2)	12	12
896	(O=C(Nc1cccs1)c2ccncn2)	10	7
897	(C(NCc1cccs1)c2occc2)	4	4
898	(O=C(NCc1ccccc1)c2cn[nH]n2)	12	12
899	(O=C(NCc1cccs1)c2cn[nH]n2)	7	7
900	(c1ccc(cc1)n2nccn2)	12	12
901	(O=C(NCCCCc1ccccc1)c2cn[nH]n2)	8	8
902	(O=C(NCCCc1ccccc1)c2cn[nH]n2)	6	6
903	(O=C(NCc1ccccn1)c2cn[nH]n2)	6	6
904	(O=C(NCc1cccnc1)c2cn[nH]n2)	7	7
905	(O=C(NCCc1ccccc1)c2cn[nH]n2)	13	13
906	(O=C(NCc1ccccc1)c2ccncn2)	9	6
907	(C1CCC(C1)c2ccccc2)	12	12
908	(C1CC(CCN1)c2nocn2)	5	5
909	(C1CC(CCN1)c2oncn2)	9	9
910	(O=C1NC=C(C2N3CC4CN2CC(C3)C4=O)C(=O)N1)	5	5
911	(C(CN1CCOCC1)C2CCCCN2)	9	9
912	(c1nncc2[nH]nnc12)	13	8
913	(c1ccc(cc1)c2nnn[nH]2)	8	8
914	(C(CN1CCCCC1)C2CCCCN2)	11	11
915	(O=S(=O)(c1ccccc1)c2cscn2)	15	13
916	(O(c1cccnn1)c2ncncn2)	18	12
917	(O=C1C2CN3CC1CN(C2)C3c4ccccn4)	5	5
918	(c1csc(c1)c2c[nH]cn2)	3	0
919	(c1ccc(cc1)c2c[nH]cn2)	6	0
920	(O=C1NN=C(Oc2ncncn2)C=C1)	11	11
921	(C1CC(CCN1)c2nnn[nH]2)	10	10
922	(N1C=NC=Nc2ccccc12)	43	23
923	(C1CC(CCN1)Oc2ccccc2)	4	4
924	(C1CCC(CC1)Nc2ocnc2)	4	4
925	(C(Nc1ocnc1)c2ccccc2)	24	13
926	(C1CCCN(CC1)c2ocnc2)	14	4
927	(N(c1ocnc1)c2ccccc2)	10	10
928	(O=C1NCNc2ccsc12)	7	5
929	(o1ccnc1\C=C\c2ccccc2)	9	9
930	(C(Cc1ccccc1)Nc2ocnc2)	13	13
931	(N(c1ccccc1)c2cn[nH]n2)	10	0
932	(O(c1ccccc1)c2cccnn2)	6	0
933	(O=C1Cc2ccccc2CN1)	10	8
934	(c1cncc(c1)c2nc[nH]n2)	17	17
935	(O=C(CCCc1oncn1)NCc2occc2)	3	3

936	(C1CCc2nocc2C1)	11	11
937	(O=C(Cc1ccccc1)NCc2oncn2)	5	5
938	(C(NC1CCCCC1)c2oncn2)	9	7
939	(o1ccnc1c2cccs2)	29	5
940	(O=C(CSC1=NC=CC(=O)N1)N2CCNCC2)	6	6
941	(O=C1NC(=NC=C1)SCCOc2ccccc2)	6	3
942	(O=C1NC(=NC=C1)Nc2ccccc2)	11	11
943	(O=C(NCc1occc1)c2ccncn2)	4	3
944	(O=C1NC2(C=CNC2=O)C=C1)	11	11
945	(O=C(N1CCNCC1)c2ccncn2)	4	4
946	(O=C(COc1ccccc1)NCc2occc2)	3	1
947	(O=C(NCc1occc1)c2ccccc2)	2	0
948	(O=C1Nc2ccccc2C1=O)	4	4
949	(C1CC2OCOC2CO1)	11	11
950	(c1ccc2snnc2c1)	22	22
951	(O=C(Nc1ccsc1)c2ccccc2)	6	5
952	(O=C1NN=Cc2c[nH]cc12)	85	85
953	(O=C(Nc1ccccc1)Nc2ccsc2)	7	4
954	(O=S(=O)(NCc1cscn1)c2ccccc2)	9	9
955	(C1SC=Cc2sccc12)	20	3
956	(O=C1NC2(CCCC2)N=C1)	12	12
957	(O=C1NCN=C1c2ccccc2)	12	12
958	(O=C1NC2(CCCCC2)N=C1)	12	12
959	(O=C(Nc1ccccc1)c2cccs2)	17	13
960	(C1CCc2c[nH]nc2CC1)	3	3
961	(C(NCc1cc[nH]c1)c2ccccc2)	30	30
962	(C(CNCc1cc[nH]c1)Cc2ccccc2)	7	7
963	(C(Cc1ccccc1)NCc2cc[nH]c2)	12	12
964	(C(NC1CCCCC1)c2cc[nH]c2)	10	10
965	(C(NC1CCCCCC1)c2cc[nH]c2)	5	5
966	(C1CCc2n[nH]cc2C1)	12	12
967	(c1cc2nc[nH]c2cn1)	111	38
968	(C(Nc1cncs1)c2ccccc2)	11	4
969	(O=C(CNS(=O)(=O)c1c[nH]cn1)Nc2ccccc2)	6	6
970	(O=C(c1ccccc1)c2ccc[nH]2)	9	9
971	(C(NCc1cccs1)c2ccccc2)	13	13
972	(O=C1CSc2ncccc2N1)	5	5
973	(O=C(CS(=O)(=O)Cc1cocn1)NCc2ccccc2)	12	12
974	(O=C1NCCc2ccccc12)	12	10
975	(O=C(N1CCCCC1)c2cccs2)	8	8
976	(c1ccn(c1)c2cccs2)	12	10

977	(O=C(NCc1ccccc1)c2cn[nH]c2)	6	6
978	(O=C1CCCN1c2nncs2)	7	3
979	(C(Cn1cccc1)c2ccccc2)	12	12
980	(C1OC=Cc2sccc12)	68	36
981	(O=C1NN=Cc2ccsc12)	6	6
982	(C1CNc2ncccc2N1)	6	6
983	(O=C(CS(=O)(=O)Cc1cocn1)N2CCNCC2)	9	9
984	(O=C(CS(=O)(=O)Cc1cocn1)NCCc2ccccc2)	12	12
985	(O=C(CS(=O)(=O)Cc1cocn1)NCCCc2ccccc2)	4	4
986	(O=C(Nc1cocc1)C23CC4CC(CC(C4)C2)C3)	7	3
987	(C(N1CCNCC1)c2oncn2)	5	5
988	(N(c1oncn1)c2oncn2)	4	1
989	(C1SC=Cc2n[nH]cc12)	1	1
990	(O=C(CNS(=O)(=O)c1cn[nH]c1)NCCc2ccccc2)	8	8
991	(O=C(CNS(=O)(=O)c1cn[nH]c1)N2CCNCC2)	8	8
992	(O=C1CN(CCC2=CCCCC2)C(=O)N1)	12	12
993	(O=C1CN(C2CCCCC2)C(=O)N1)	12	12
994	(o1cc2ccccc2n1)	11	4
995	(O=C1CN(Cc2ccccc2)C(=O)N1)	5	5
996	(O=C1CN(CCc2cccs2)C(=O)N1)	12	12
997	(c1cc2sccc2cn1)	38	10
998	(O=C(CCCc1ccon1)NCCc2ccccc2)	5	2
999	(O=C1NC(=O)N2CSCC12)	7	7
1000	(O=C(NCc1nnc[nH]1)C23CC4CC(CC(C4)C2)C3)	8	8
1001	(O=C1CCCc2[nH]ccc12)	60	7
1002	(O=C(CNS(=O)(=O)C1=CNC(=O)NC1=O)Nc2ccccc2)	12	12
1003	(O=C(CNS(=O)(=O)C1=CNC(=O)NC1=O)NCc2ccccc2)	9	9
1004	(O=C(CS(=O)(=O)Cc1cocn1)NCCCN2CCNCC2)	4	4
1005	(O=C(CS(=O)(=O)Cc1cocn1)Nc2ccccc2)	12	12
1006	(O=C(NCCCN1CCNCC1)C2CCNCC2)	3	3
1007	(O=C(Cn1ccnn1)Nc2ccccc2)	12	12
1008	(C(=C\c1nc[nH]n1)/c2ccccc2)	7	2
1009	(O=C(CSc1nnn[nH]1)NCCc2ccccc2)	6	6
1010	(O=C(OCc1ccccc1)c2cn[nH]n2)	7	7
1011	(O=C1NC(C=C1)c2ccccc2)	9	3
1012	(O=C(Nc1ccccc1)C2=CC(=O)NC(=O)N2)	1	1
1013	(C1NC=Nc2nccn12)	3	3
1014	(O=C(Nc1ncncn1)NS(=O)(=O)Oc2ccccc2)	5	5
1015	(O=C(NC1NC(=O)NC1=O)c2ccccc2)	7	7
1016	(O=C(Nc1ccccc1)\C=C\c2cc[nH]c2)	5	2
1017	(N=C1NC(=CS1)c2ccccc2)	3	3

1018	(O=S(=O)(Nc1ccccc1)c2ccccc2)	12	7
1019	(O=C(CNS(=O)(=O)c1ccccc1)NCCc2ccccc2)	10	8
1020	(O=S(=O)(NCCSc1nnn[nH]1)c2ccccc2)	5	5
1021	(O=C1C=CN=C2NC=NN12)	1	1
1022	(O=C(CNS(=O)(=O)c1ccccc1)NCCSc2ccccc2)	10	7
1023	(c1ccc(nc1)c2cccs2)	13	0
1024	(c1ccc(cc1)c2ccccn2)	23	5
1025	(O=C(Nc1ccccc1)\C=C/c2ccccc2)	1	0
1026	(O=C(NCc1ccccc1)C(=O)Nc2ccccc2)	10	10
1027	(O=C(CCN1CCNCC1)Nc2ccccc2)	8	8
1028	(C(=C/c1nccs1)/c2ccccc2)	7	0
1029	(o1cnc(\C=C\c2ccccc2)n1)	5	5
1030	(C1CCc2ccoc2C1)	12	6
1031	(O=C(CNc1ccccc1)NCCSc2ccccc2)	8	8
1032	(O=C(NCCNCc1ccccc1)c2cnon2)	6	6
1033	(O=C1C=CC=C2OC=CN12)	6	6
1034	(O=C(CSc1ncccn1)NCCc2ccccc2)	5	0
1035	(O=C(CSc1ncccn1)N2CCCCC2)	8	4
1036	(o1cccc1c2ccncn2)	22	17
1037	(O=C1CNC=C2CC=CC=C2N1)	4	4
1038	(O=C1CNC2=CC=CCC2=CN1)	2	2
1039	(O=C(CSc1ncccn1)Nc2cccs2)	5	0
1040	(O=C1NC(=CC(=O)N1)Nc2ccccc2)	2	2
1041	(C1Cc2ccncc2CO1)	5	5
1042	(C(Nc1ncn[nH]1)c2occc2)	4	4
1043	(C(Nc1ncn[nH]1)c2ccccc2)	12	2
1044	(O=C(c1occc1)n2cncn2)	5	4
1045	(O=S(=O)(Nc1ccon1)c2ccccc2)	6	6
1046	(O=C1OC=Nc2ccccc12)	6	5
1047	(O=C(CSc1ncccn1)NCc2ccccc2)	12	0
1048	(O=C(CSc1nc[nH]n1)Nc2ccccc2)	3	3
1049	(C1Cc2cn[nH]c2C1)	8	8
1050	(O=S(=O)(Nc1ccncn1)c2ccccc2)	15	15
1051	(O=C(CCSc1ncccn1)Nc2ccccc2)	10	1
1052	(O=C1CCSc2ncnn12)	10	10
1053	(C1CCc2ncncc2C1)	8	0
1054	(C1C=COc2ccccc12)	8	5
1055	(O=C1OC=Nc2sccc12)	6	5
1056	(O=C(Nc1cccs1)c2ccccc2)	9	3
1057	(C1Cc2ccccc2CO1)	4	4
1058	(O=C1CCSC2=NC=CCN12)	6	4

1059	(O=S(=O)(Nc1oncc1)c2ccccc2)	10	10
1060	(O=C(OCc1ccccc1)C2=CNC(=O)CC2)	8	8
1061	(O=C1NC(=O)C(Nc2ccccc2)S1)	9	9
1062	(C(c1ccccc1)c2ccccc2)	6	3
1063	(S(c1ccccc1)c2cn[nH]c2)	24	3
1064	(O=C1NNC=C1CC2=CNNC2=O)	3	3
1065	(O=C1NC(=O)N2C=CSC2=N1)	5	0
1066	(O=C(CCCC(=O)OCC(=O)c1ccccc1)Nc2ccccc2)	5	3
1067	(O=C(COc1ccccc1)N2CCNCC2)	10	10
1068	(O=C1OC=CC2=C1NCCC2)	4	4
1069	(O=C1CCCc2occc12)	6	5
1070	(O=C(CNC(=O)C1CCCCC1)OCC(=O)c2ccccc2)	5	4
1071	(C(c1cn[nH]c1)c2cn[nH]c2)	9	6
1072	(O=C1NCC=C1Nc2ccccc2)	8	8
1073	(C1CCCc2ncccc2CC1)	3	3
1074	(O=C(Nc1ccccc1)C2=CNC(=O)NC2)	7	7
1075	(O=C1NC(=O)C2=C(NC(=O)N2)N1)	1	1
1076	(O=C(NCCCN1CCOCC1)C(=O)Nc2ccccc2)	6	6
1077	(O=C(CCNCc1cccnc1)Nc2ccccc2)	2	2
1078	(O=C(Nc1ccccc1)C2CC(=O)N=CS2)	3	3
1079	(N(c1ccccc1)c2cccnn2)	10	0
1080	(N1C=CSc2ccccc12)	7	5
1081	(O=C(CNS(=O)(=O)c1ccccc1)N2CCNCC2)	5	5
1082	(C(COc1ccccc1)CN2CCNCC2)	11	11
1083	(O=C(CNS(=O)(=O)c1ccccc1)N2CCCCC2)	5	5
1084	(O=C(CNS(=O)(=O)c1ccccc1)NCCSCc2ccccc2)	6	4
1085	(O(c1ccccc1)c2ccccc2)	6	3
1086	(O=C(COC(=O)c1ccccc1)c2ccccc2)	2	2
1087	(O=C(CNS(=O)(=O)c1ccccc1)Nc2cccnc2)	5	5
1088	(O=C(CCN1CCCCC1)Nc2ccccc2)	10	10
1089	(O=C(CNCCc1ccccc1)NCc2ccccc2)	5	5
1090	(O=C(COc1ccccc1)NS(=O)(=O)c2ccccc2)	5	1
1091	(O=C1C=CC2=C1CSC=CN2)	1	0
1092	(O=S(=O)(CCCSc1ccccn1)Cc2ccccc2)	5	0
1093	(O=C(NCc1occc1)C(=O)Nc2ccccc2)	6	6
1094	(O=C1NCNc2sccc12)	5	3
1095	(C=C(NC(=O)c1ccccc1)C(=O)NC2CCCCC2)	3	1
1096	(O=C(OC1CCCCC1)c2ccccc2)	6	1
1097	(O=C(Nc1ccccc1)Nc2ccccn2)	6	6
1098	(O=C(Nc1ccccc1)\C=C/c2occc2)	1	0
1099	(O=C(CSc1ncn[nH]1)Nc2ccccc2)	1	1

1100	(O=C(COc1ccccc1)Nc2nc[nH]n2)	1	1
1101	(C1CN=C(O1)c2ccc[nH]2)	2	1
1102	(C1Cc2[nH]ncc2C=N1)	1	0
1103	(c1ccc(cc1)c2cnc[nH]2)	22	16
1104	(O=C(CSc1c[nH]cn1)NCC2CCCO2)	11	9
1105	(O=C(CCc1oncn1)NCc2ccccc2)	5	5
1106	(O=C1NC=Cc2cc[nH]c12)	34	24
1107	(O=C1OC=Cc2ncccc12)	6	6
1108	(O=C(CSc1c[nH]cn1)Nc2ccccc2)	12	0
1109	(O=C(CN1CCNC1=O)NCCc2ccccc2)	6	6
1110	(O=C1C=Cc2ccccc12)	15	0
1111	(C(Cc1ccccc1)NCc2ocnn2)	10	0
1112	(O=C1CC(CN1)c2ocnn2)	10	10
1113	(O=C(CSc1ocnn1)N2CCCCC2)	12	12
1114	(O=C(OC1CCCCCC1)C2=CNC(=O)CC2)	9	9
1115	(O=C1CC(C=CN1)c2ccccc2)	7	7
1116	(O=C(OCC1CCCCC1)C2=CNC(=O)CC2)	6	6
1117	(O=C1NC=C(C(=O)N1)S(=O)(=O)N2CCCCC2)	8	8
1118	(O=C(NC1=CNNC1=O)Nc2ccccc2)	12	12
1119	(O=C(Nc1ccccc1)C2CC2)	9	9
1120	(O=C1NC(=O)C2=NCCN=C2N1)	6	6
1121	(O=C1NC(=O)c2nccnc2N1)	3	3
1122	(C1Cc2cnoc2C=C1)	22	13
1123	(O=C(Nc1cccnc1)c2ccon2)	7	7
1124	(O=C(NC1=CNNC1=O)c2ccon2)	10	10
1125	(O=C(N1CCCCC1)c2ccon2)	8	8
1126	(O=C(NCCc1ccccc1)c2ccon2)	7	7
1127	(O=C(Nc1cc[nH]n1)c2ccon2)	6	1
1128	(O=C(Nc1ccn[nH]1)c2ccon2)	5	4
1129	(O=C(Nc1cn[nH]c1)c2ccon2)	19	19
1130	(O=C(CCS(=O)(=O)c1cccs1)Nc2ccccc2)	19	19
1131	(O=C(CCNS(=O)(=O)c1cccs1)Nc2ccccc2)	4	4
1132	(C(c1cocn1)n2ccnn2)	8	8
1133	(c1cc2cnccn2n1)	4	0
1134	(c1csc(n1)c2nccs2)	7	0
1135	(O=C1NC=Cn2cnnc12)	5	5
1136	(O=S(=O)(Nc1ccccc1)c2ccn[nH]2)	1	1
1137	(C(c1ccccc1)c2cncnc2)	1	1
1138	(O=C1NC(=O)c2[nH]ncc2N1)	15	15
1139	(O=C(Cn1ccnn1)NC(=O)Nc2ccccc2)	7	7
1140	(O=C(CS(=O)(=O)c1ncc[nH]1)Nc2ccccc2)	10	3

1141	(O=C(CNC(=O)C1CCCCC1)Nc2ccccc2)	12	12
1142	(c1cn2ccnc2cn1)	10	8
1143	(O=C(CSc1ocnc1)Nc2ccccc2)	9	1
1144	(O=C(CSc1ocnc1)NC2CCCCC2)	7	0
1145	(O=C(CSc1ocnc1)c2ccccc2)	7	0
1146	(C(Cc1ccccc1)Nc2ncccn2)	8	0
1147	(C(Nc1ncccn1)c2ccccc2)	11	0
1148	(O=C(Cc1cccs1)NCC(=O)Nc2ccccc2)	4	3
1149	(O=C(CCCS(=O)(=O)c1ncccn1)Nc2ccccc2)	4	2
1150	(O=C(CNCc1ccccc1)NC2CCCCC2)	4	1
1151	(O=C(NC1N=CNC1=O)c2ccccc2)	8	1
**All**		**41294**	**32972**
